# Supplementary material for: Synthesis and Characterization of a New Norfloxacin/Resorcinol Cocrystal with Enhanced Solubility and Dissolution Profile
Source: Pharmaceutics. 2021 Dec 27;14(1):49. doi: 10.3390/pharmaceutics14010049 (PMC8778133; doi:10.3390/pharmaceutics14010049)
Supplement: Supplementary file 1 [file pharmaceutics-14-00049-s001.zip › pharmaceutics-1494689-supplementary.pdf]

pharmaceutics

MDPI

Supplementary Materials: Synthesis and Characterization of a New Norfloxacin/Resorcinol Cocrystal with Enhanced Solubility and Dissolution Profile

1. Characterization of Norfloxacin initial batch and its resorcinol cocrystals

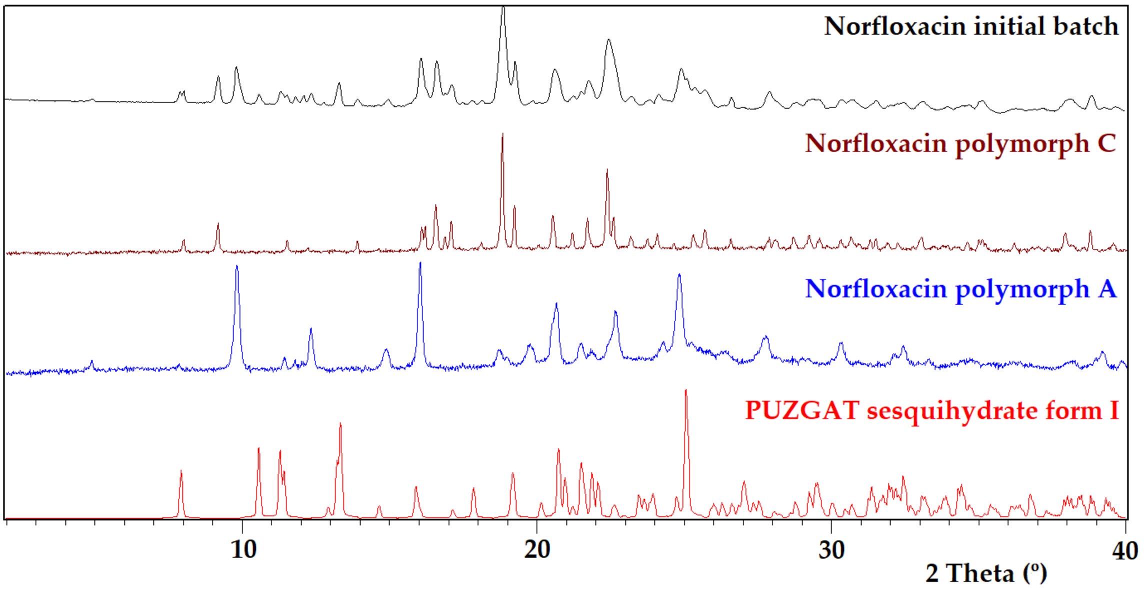

Figure S1. XRPD of Norfloxacin initial batch.

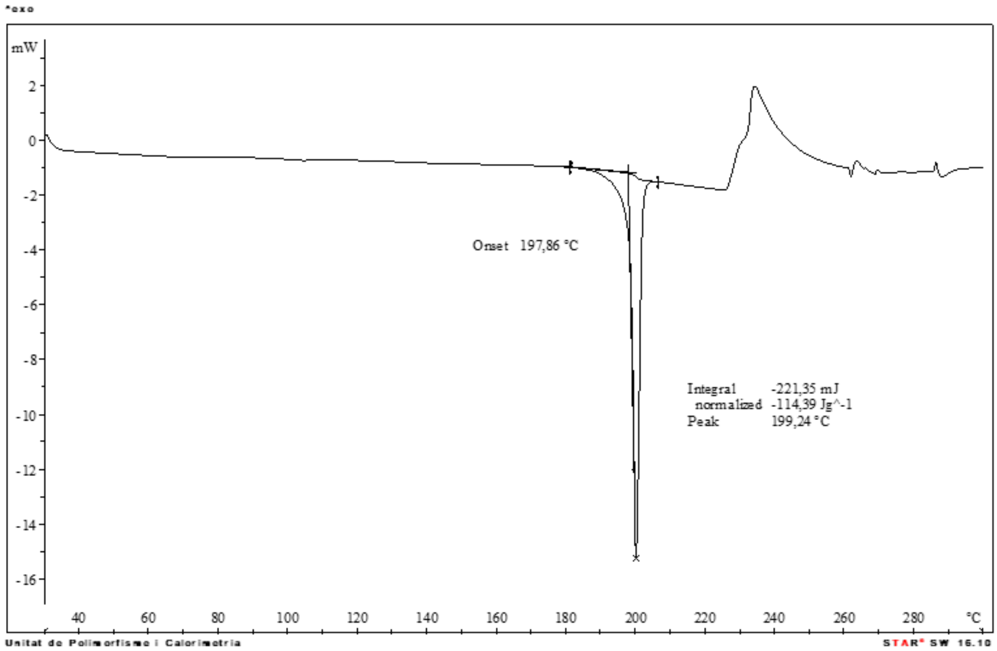

Figure S2. DSC of Norfloxacin – resorcinol cocrystal.

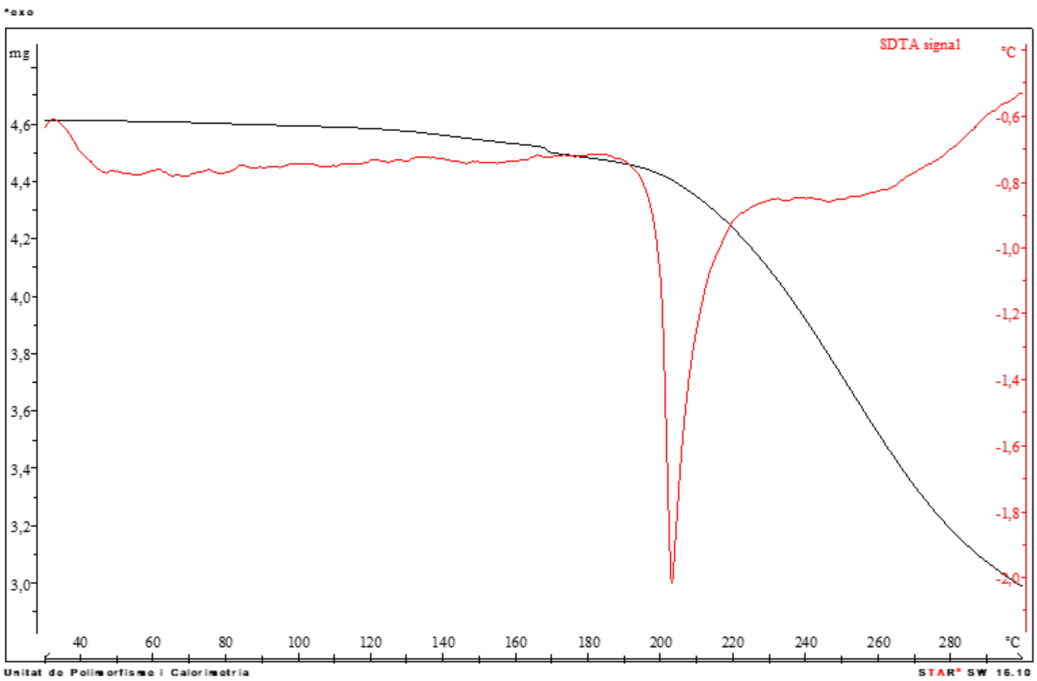

Figure S3. TGA of Norfloxacin – resorcinol cocrystal.

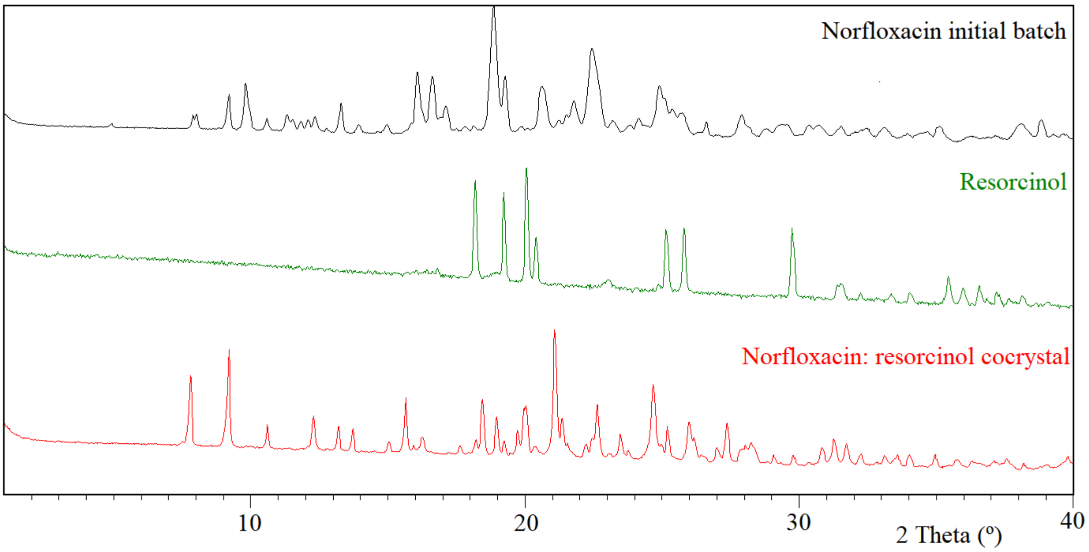

Figure S4. XRPD of Norfloxacin – resorcinol cocrystal.

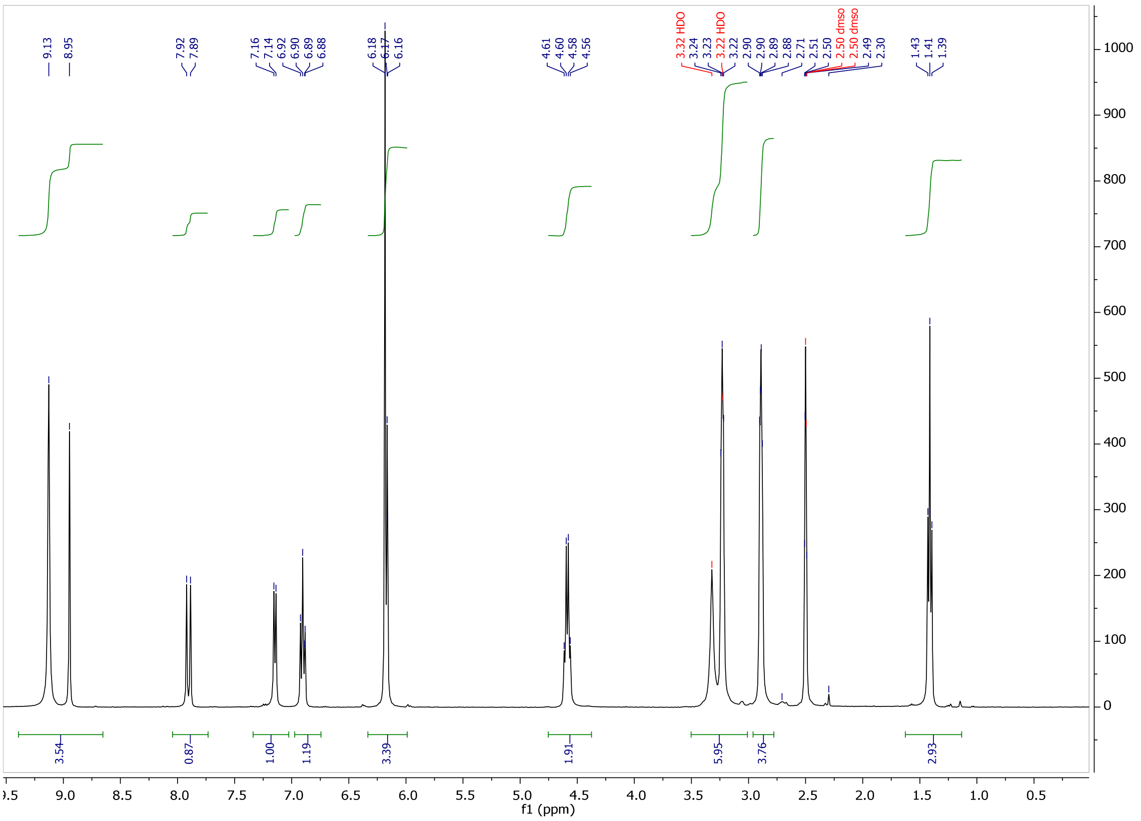

Figure S5. ^{1}H-NMR (dmso-d_6/delay: 1 second /pulse: 45°/scans: 32) of Norfloxacin – resorcinol cocrystal.

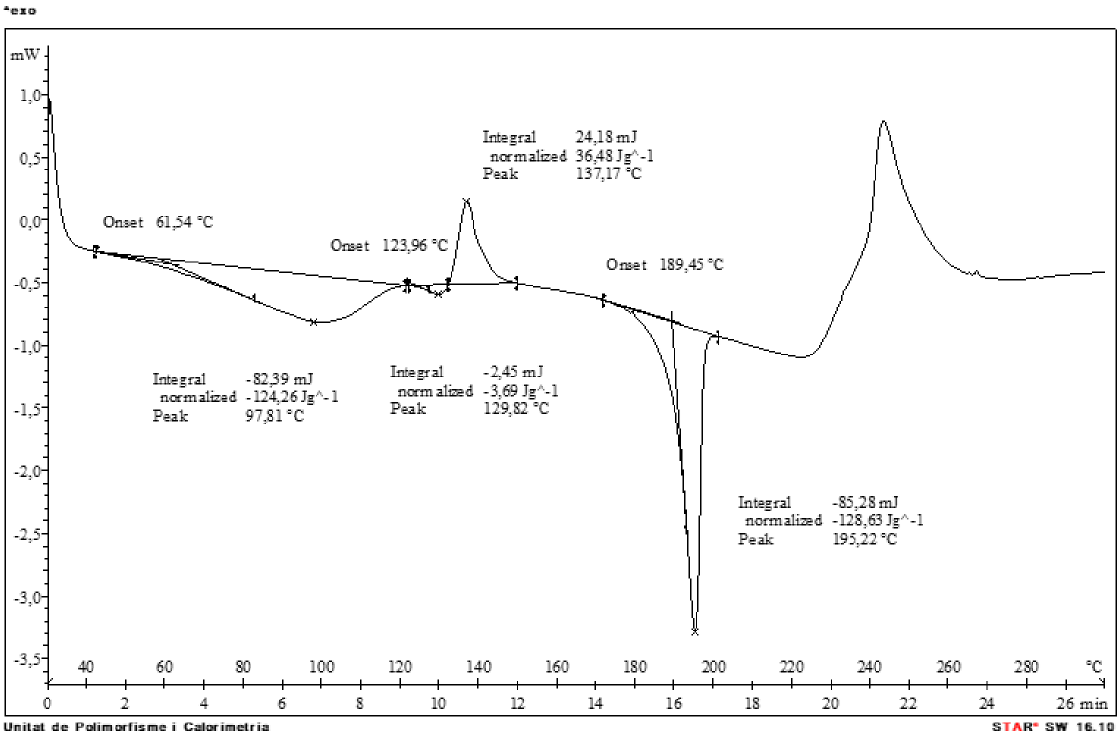

Figure S6. DSC of Norfloxacin – resorcinol cocrystal monohydrate .

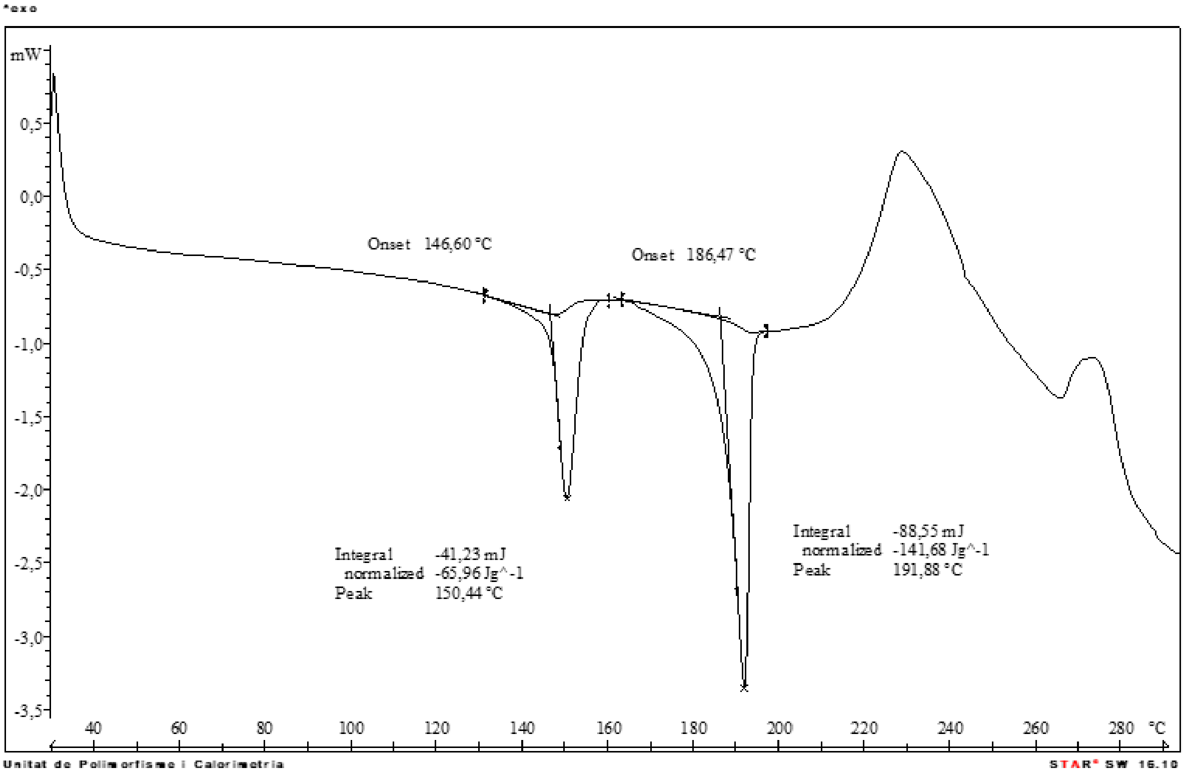

Figure S7. DSC (crucible without hole) of Norfloxacin – resorcinol cocrystal monohydrate.

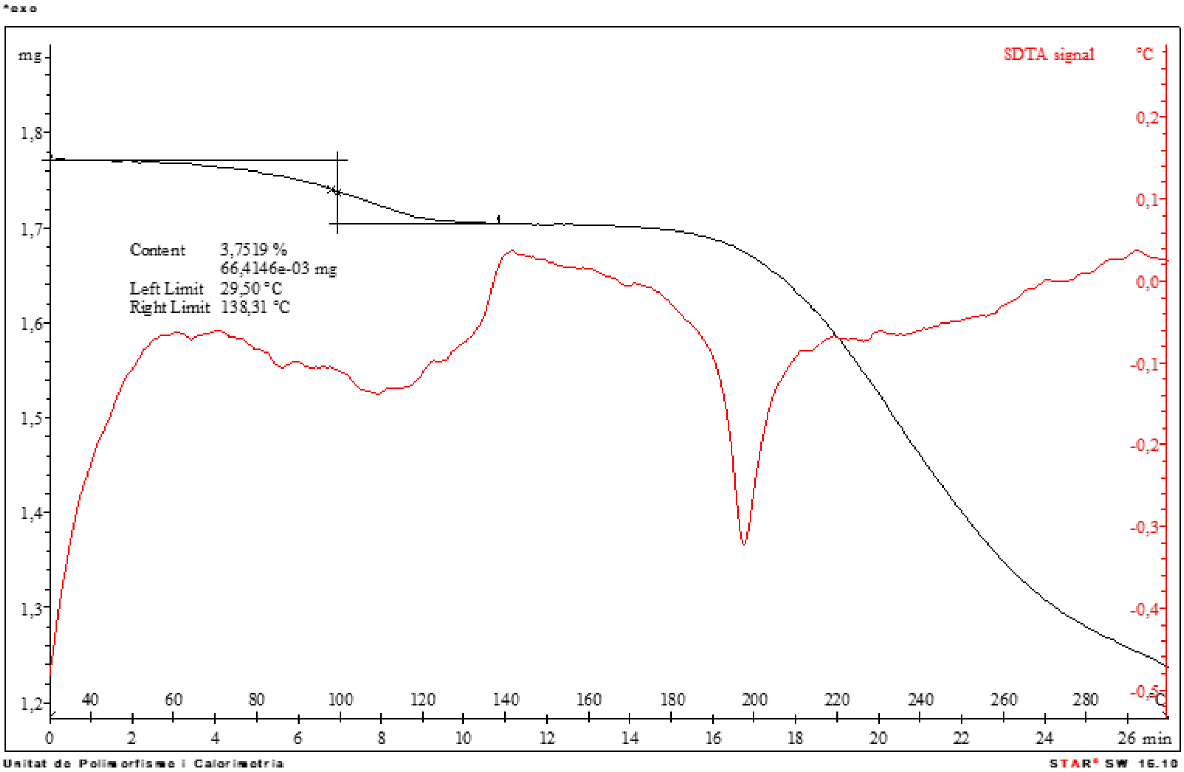

Figure S8. TGA of Norfloxacin – resorcinol cocrystal monohydrate.

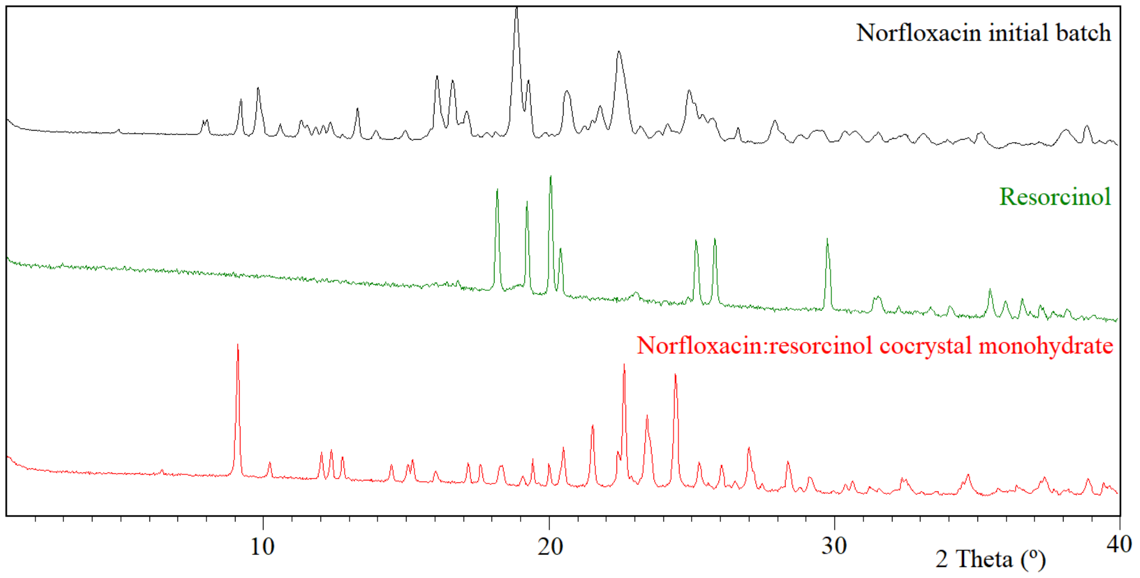

Figure S9. XRPD of Norfloxacin – resorcinol cocrystal monohydrate.

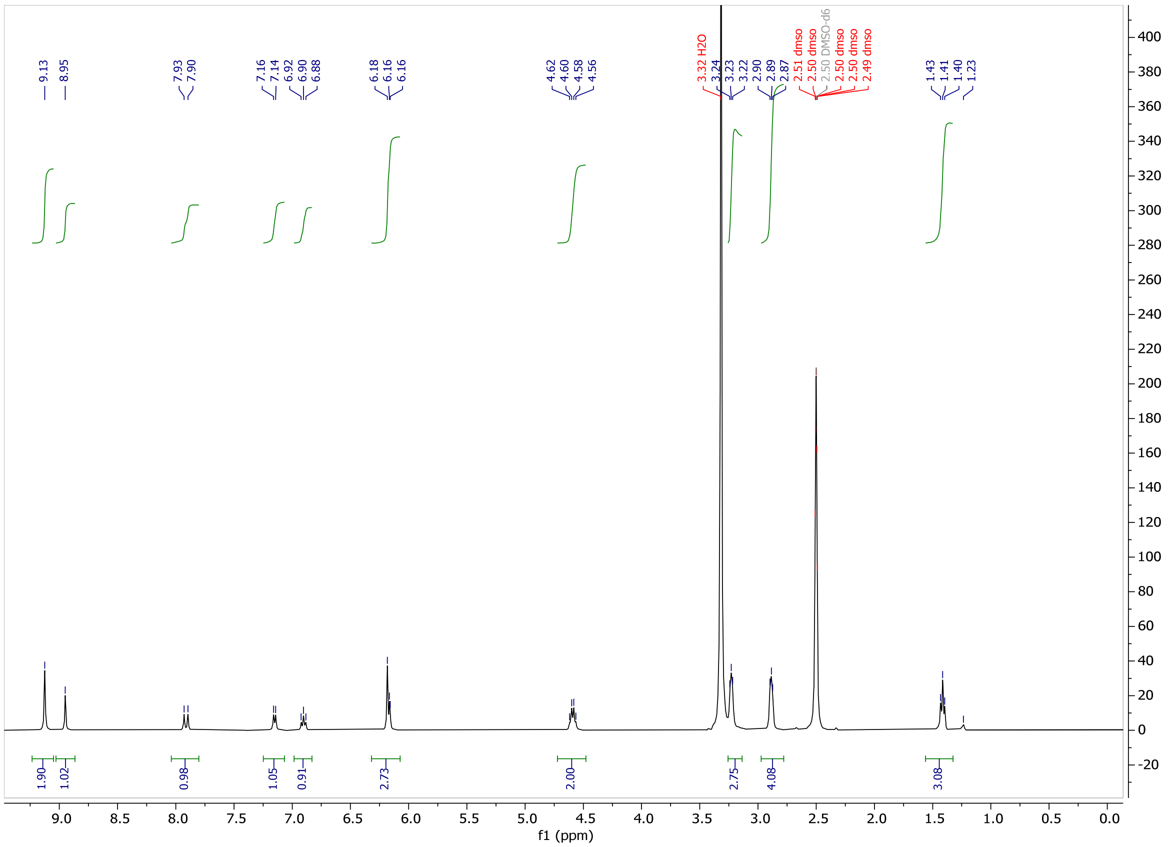

Figure S10. ^{1}H-NMR (dmso-d_6/delay: 1second /pulse: 45°/scans: 16) of Norfloxacin - resorcinol cocrystal monohydrate.

2. Norfloxacin and Resorcinol Molar Extinction Coefficients

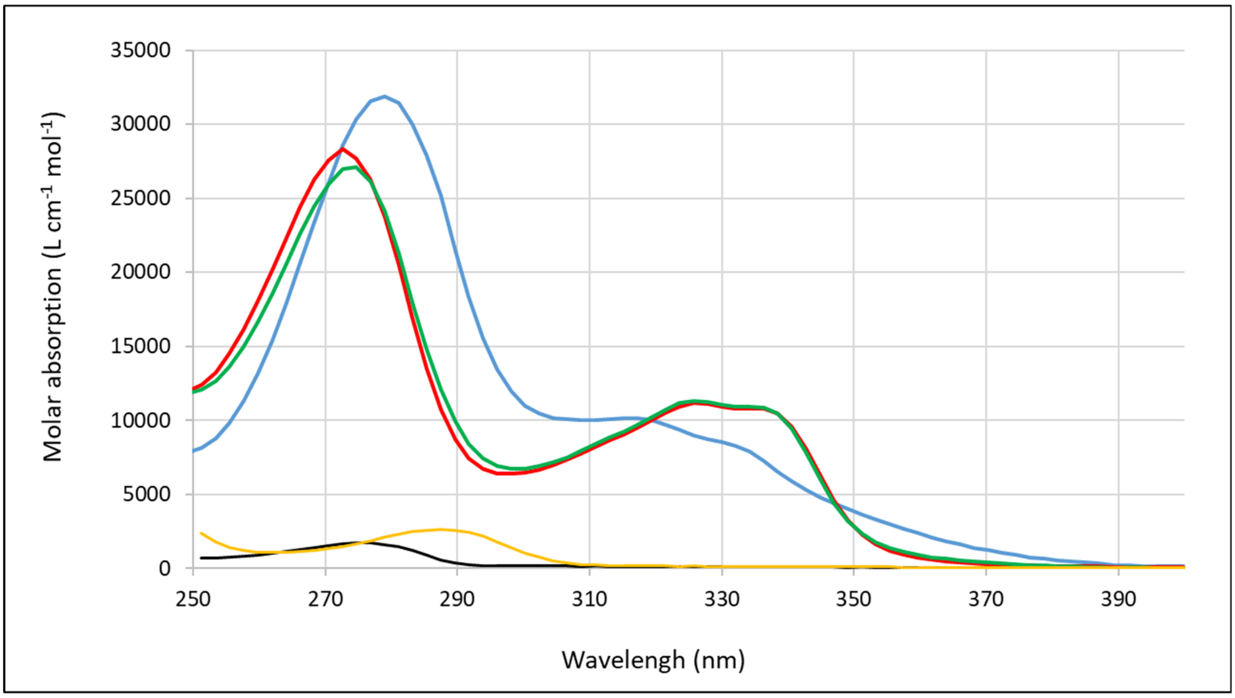

Figure S11. MEC absorption profiles of the different ionic species of Norfloxacin and Resorcinol: H_2Nor^+ (—); $HNor^{\pm}$ (—); Nor^- (—); H_2Res (—); $HRes^-$ (—).

3. Norfloxacin species distribution diagram

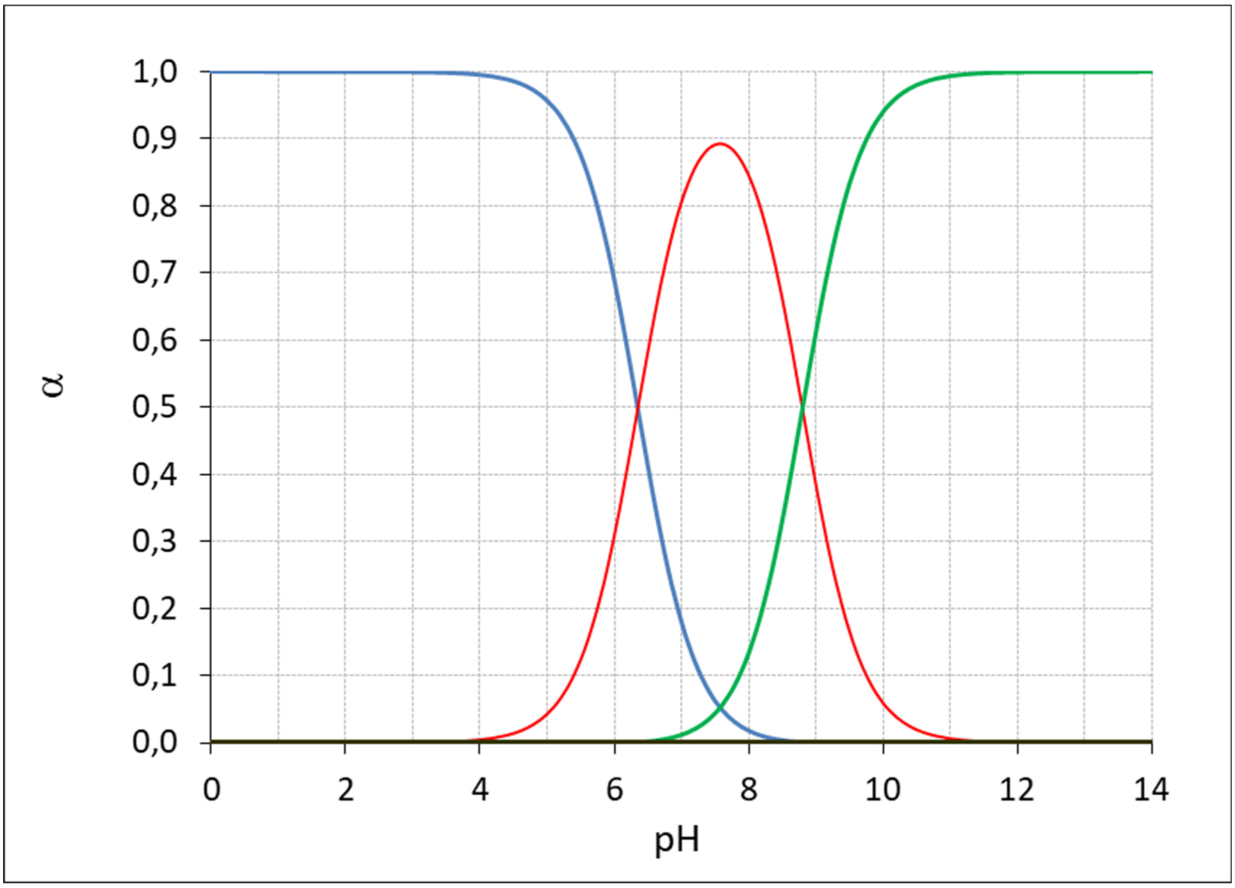

Figure S12. Norfloxacin species distribution diagram: H_2Nor^+ (—); $HNor^{\pm}$ (—); Nor^- (—).

4. Characterization of Norfloxacin tetrahydrate form

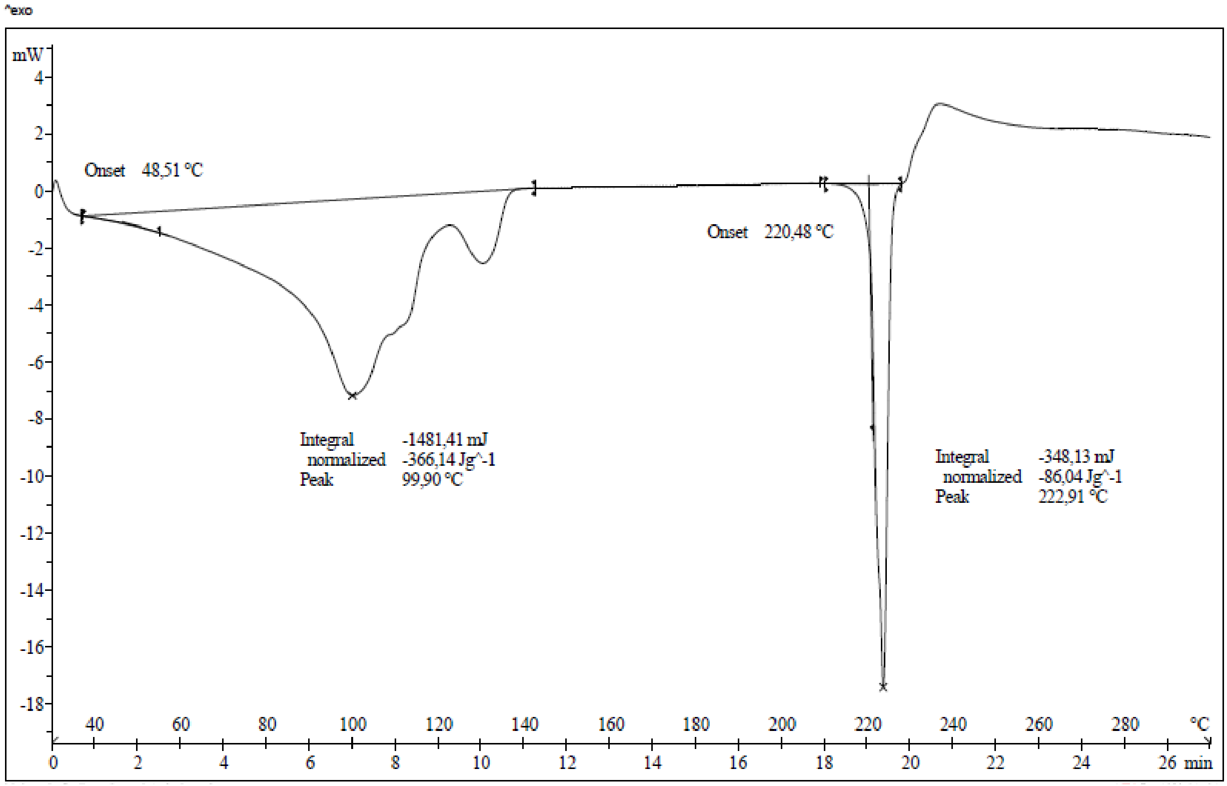

Figure S13. DSC of Norfloxacin tetrahydrate form.

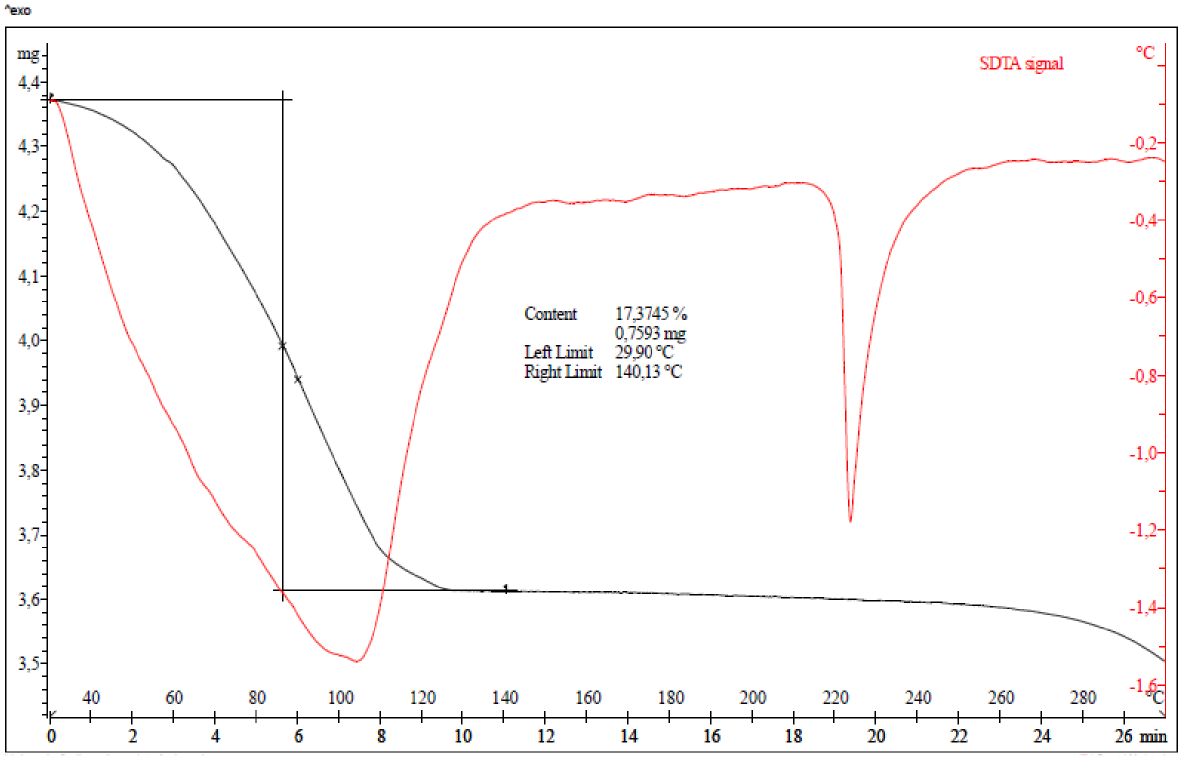

Figure S14. TGA of Norfloxacin tetrahydrate form: a weight loss of 17.4% is detected from 30 °C to 140 °C which could be attributed to 4 molecules of water per one molecule of Norfloxacin (theoretical weight loss of 17.6%).

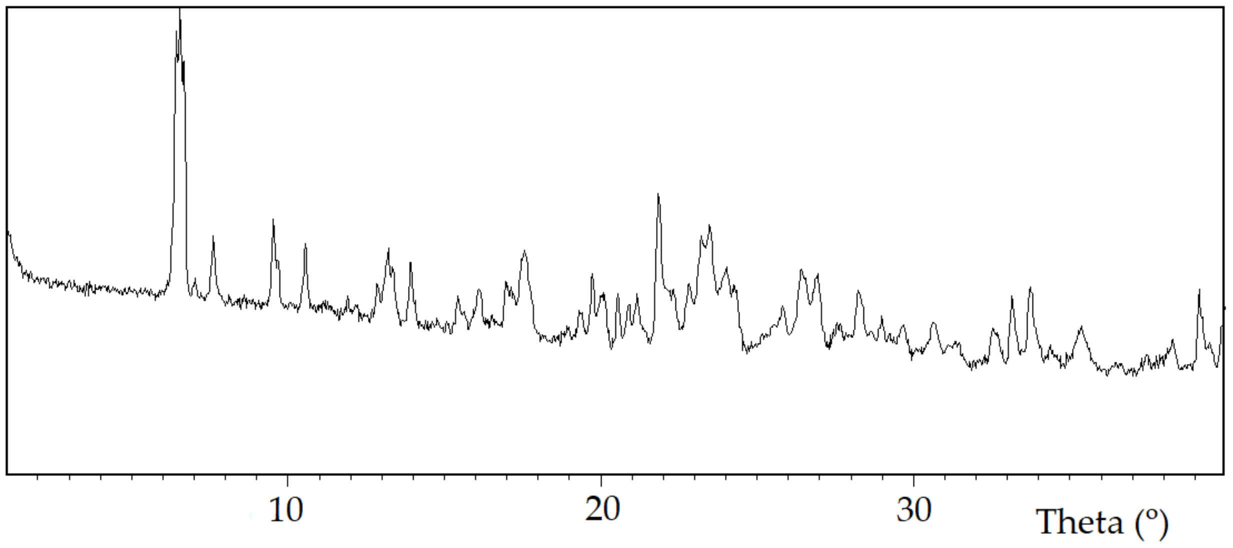

Figure S15. XRPD of Norfloxacin tetrahydrate form.

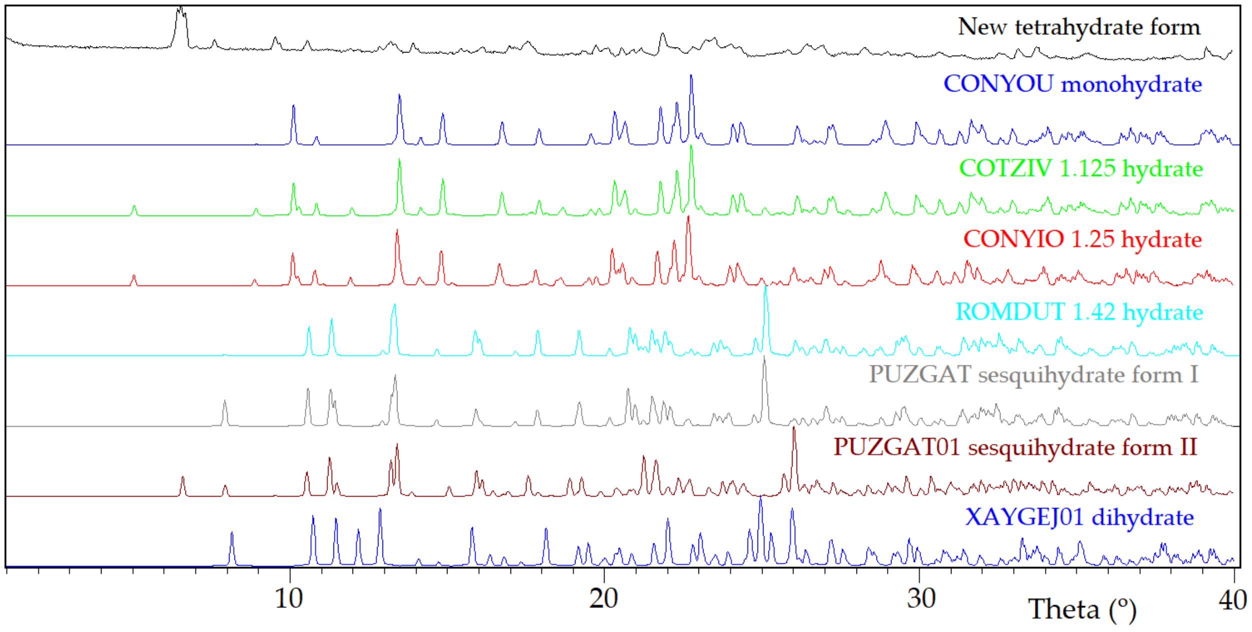

Figure S16. Comparative XRPD diffractograms of Norfloxacin tetrahydrate form (black) and simulated from the cif: monohydrate (CONYOU, blue) [1], 1.125 hydrate (COTZIV, green) [1], 1.25 hydrate (CONYIO, red) [1], 1.42 hydrate (ROMDUT, light blue) [2], sesquihydrate form I (PUZGAT, grey) [3], sesquihydrate form II (PUZGAT01, brown) [3] and dihydrate (XAYGEJ01, dark blue) [4].

5. XRPD of the new multicomponent forms of Norfloxacin

The XRPD characteristic peaks of the new forms of Norfloxacin are included in Table S1.

Table S1. Characteristic 2Theta peaks (°) of the new forms of Norfloxacin.

Norfloxacin resorcinol cocrystal		Norfloxacin resorcinol cocrystal monohydrate	
Pos. [°2Th.]	Rel. Int. [%]	Pos. [°2Th.]	Rel. Int. [%]
7.8059	46.58	9.1209	75.37
9.2018	72.24	10.2392	3.95
10.6092	10.34	12.0623	6.68
12.2935	16.06	12.3965	12.19
13.1946	10.61	12.8007	8.03
13.7225	9.35	14.5179	4.47
15.658	29.44	15.087	5.46
18.4556	28.74	15.2627	6.66
18.9756	17.06	17.2056	6
19.7401	9.97	17.6454	5.07
19.9502	23.11	18.4232	4.87
20.0678	22.3	19.4757	6.5
21.0941	100	20.0528	5.33
21.37	16.83	20.5515	10.5
22.6395	26.1	21.5662	21.54
23.4864	9.02	22.4607	11.01
24.6803	42.16	22.6753	100
25.2092	10.81	23.4653	36.23
25.9875	15.91	23.6142	18.53
27.3743	15.46	24.4836	80.12
30.8358	5.05	25.3098	5.99
31.2678	8.43	26.0984	6.41
31.7145	6.51	27.0588	13.69
34.9451	3.15	28.4384	6.58

6. References

1. Roy, S.; Goud, N. R.; Babu, N. J.; Iqbal, J.; Kruthiventi, A. K.; Nangia, A. Crystal Structures of Norfloxacin Hydrates. *Cryst. Growth Des*. **2008**, *8*, 4343-4346.
2. Ravindra, N.V.; Panpalia, G. M.; Jagarlapudi, A. R. Norfloxacin sesquihydrate. *Acta Crystallogr Sect E*. **2009**, *65*, o303.
3. Puigjaner, C.; Barbas, R.; Portell, A.; Font-Bardia, M.; Alcobé, X.; Prohens, R. Revisiting the Solid State of Norfloxacin. *Cryst. Growth Des*. **2010**, *10*, 2948-2953.
4. Holstein, J. J.; Hübschle, C. B.; Dittrich, B. Electrostatic properties of nine fluoroquinolone antibiotics derived directly from their crystal structure refinaments. *CrystEngComm*, **2012**, *14*, 2520- 2531.
